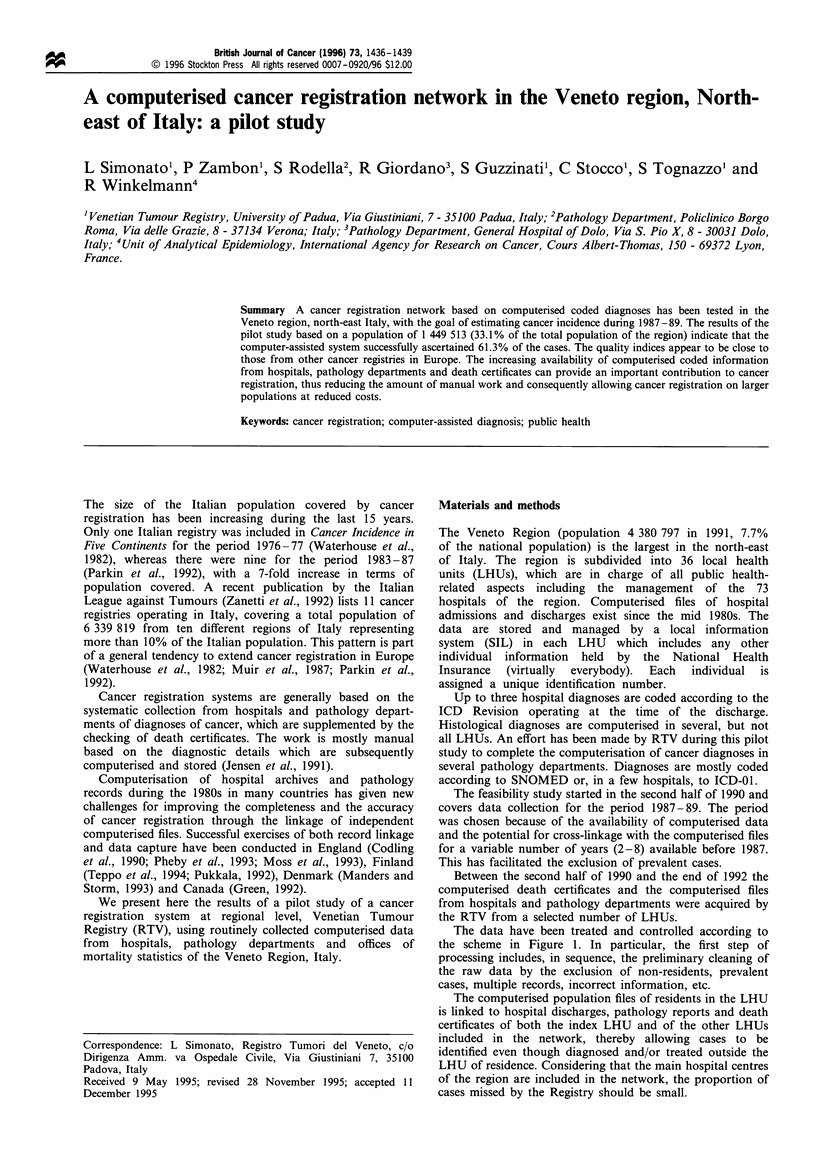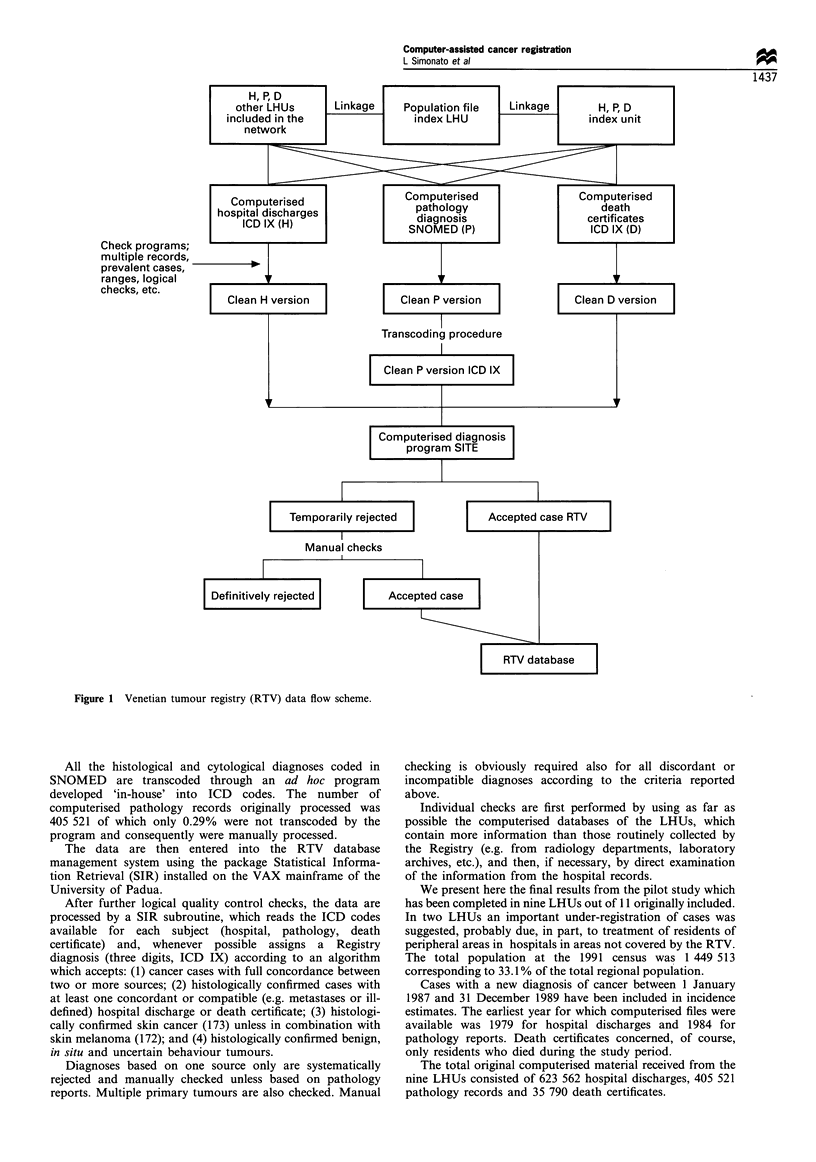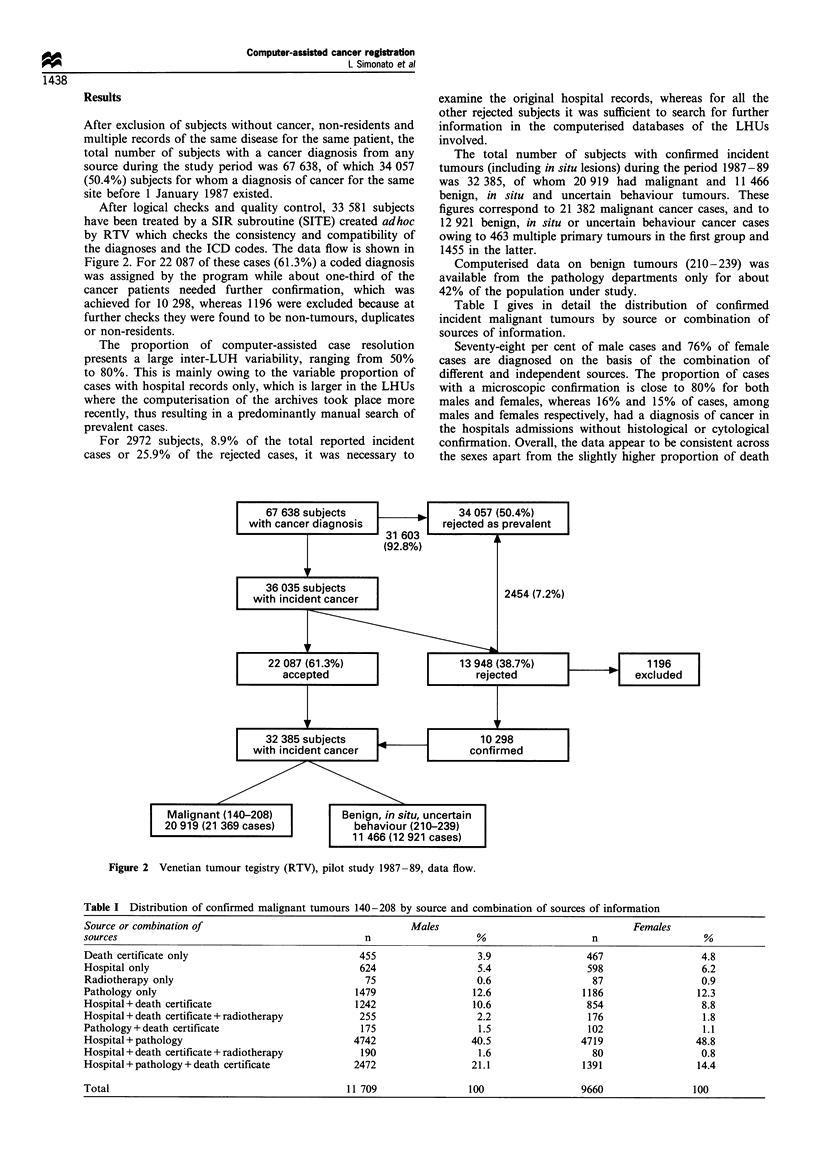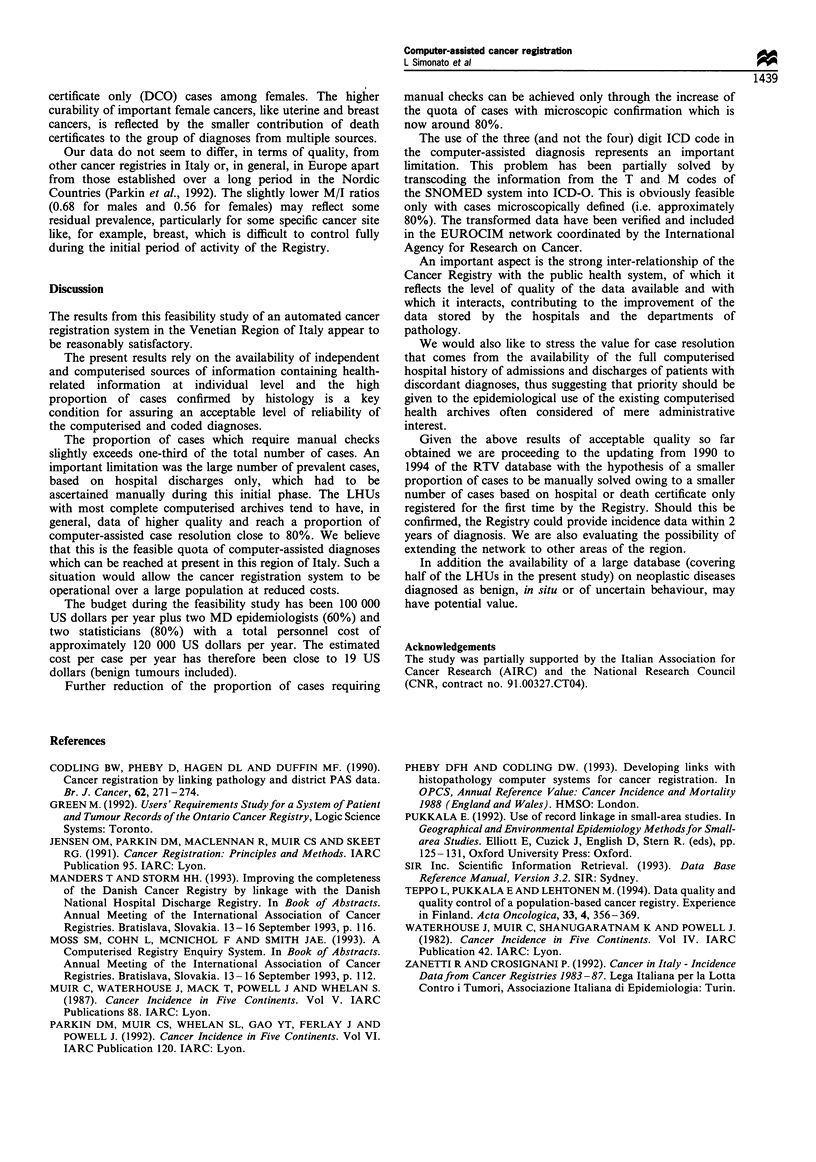# A computerised cancer registration network in the Veneto region, north-east of Italy: a pilot study.

**DOI:** 10.1038/bjc.1996.273

**Published:** 1996-06

**Authors:** L. Simonato, P. Zambon, S. Rodella, R. Giordano, S. Guzzinati, C. Stocco, S. Tognazzo, R. Winkelmann

**Affiliations:** Venetian Tumour Registry, University of Padua, Italy.

## Abstract

A cancer registration network based on computerised coded diagnoses has been tested in the Veneto region, north-east Italy, with the goal of estimating cancer incidence during 1987-89. The results of the pilot study based on a population of 1,449,513 (33.1% of the total population of the region) indicate that the computer-assisted system successfully ascertained 61.3% of the cases. The quality indices appear to be close to those from other cancer registries in Europe. The increasing availability of computerised coded information from hospitals, pathology departments and death certificates can provide an important contribution to cancer registration, thus reducing the amount of manual work and consequently allowing cancer registration on larger populations at reduced costs.


British Journal of Cancer (1996) 73, 1436-1439
© 1996 Stockton Press All rights reserved 0007-0920/96 $12.00

### A computerised cancer registration network in the Veneto region, North-east of Italy: a pilot study

L Simonato[1], P Zambon[1], S Rodella[2], R Giordano[3], S Guzzinati[1], C Stocco[1], S Tognazzo[1] and R Winkelmann[4]

[1]*Venetian Tumour Registry, University of Padua, Via Giustiniani, 7 - 35100 Padua, Italy;* [2]*Pathology Department, Policlinico Borgo Roma, Via delle Grazie, 8 - 37134 Verona; Italy;* [3]*Pathology Department, General Hospital of Dolo, Via S. Pio X, 8 - 30031 Dolo, Italy;* [4]*Unit of Analytical Epidemiology, International Agency for Research on Cancer, Cours Albert-Thomas, 150 - 69372 Lyon, France.*

**Summary** A cancer registration network based on computerised coded diagnoses has been tested in the Veneto region, north-east Italy, with the goal of estimating cancer incidence during 1987-89. The results of the pilot study based on a population of 1 449 513 (33.1% of the total population of the region) indicate that the computer-assisted system successfully ascertained 61.3% of the cases. The quality indices appear to be close to those from other cancer registries in Europe. The increasing availability of computerised coded information from hospitals, pathology departments and death certificates can provide an important contribution to cancer registration, thus reducing the amount of manual work and consequently allowing cancer registration on larger populations at reduced costs.

**Keywords:** cancer registration; computer-assisted diagnosis; public health

The size of the Italian population covered by cancer registration has been increasing during the last 15 years. Only one Italian registry was included in *Cancer Incidence in Five Continents* for the period 1976-77 (Waterhouse *et al.*, 1982), whereas there were nine for the period 1983-87 (Parkin *et al.*, 1992), with a 7-fold increase in terms of population covered. A recent publication by the Italian League against Tumours (Zanetti *et al.*, 1992) lists 11 cancer registries operating in Italy, covering a total population of 6 339 819 from ten different regions of Italy representing more than 10% of the Italian population. This pattern is part of a general tendency to extend cancer registration in Europe (Waterhouse *et al.*, 1982; Muir *et al.*, 1987; Parkin *et al.*, 1992).

Cancer registration systems are generally based on the systematic collection from hospitals and pathology departments of diagnoses of cancer, which are supplemented by the checking of death certificates. The work is mostly manual based on the diagnostic details which are subsequently computerised and stored (Jensen *et al.*, 1991).

Computerisation of hospital archives and pathology records during the 1980s in many countries has given new challenges for improving the completeness and the accuracy of cancer registration through the linkage of independent computerised files. Successful exercises of both record linkage and data capture have been conducted in England (Codling *et al.*, 1990; Pheby *et al.*, 1993; Moss *et al.*, 1993), Finland (Teppo *et al.*, 1994; Pukkala, 1992), Denmark (Manders and Storm, 1993) and Canada (Green, 1992).

We present here the results of a pilot study of a cancer registration system at regional level, Venetian Tumour Registry (RTV), using routinely collected computerised data from hospitals, pathology departments and offices of mortality statistics of the Veneto Region, Italy.

Correspondence: L Simonato, Registro Tumori del Veneto, c/o Dirigenza Amm. va Ospedale Civile, Via Giustiniani 7, 35100 Padova, Italy
Received 9 May 1995; revised 28 November 1995; accepted 11 December 1995

### Materials and methods

The Veneto Region (population 4 380 797 in 1991, 7.7% of the national population) is the largest in the north-east of Italy. The region is subdivided into 36 local health units (LHUs), which are in charge of all public health-related aspects including the management of the 73 hospitals of the region. Computerised files of hospital admissions and discharges exist since the mid 1980s. The data are stored and managed by a local information system (SIL) in each LHU which includes any other individual information held by the National Health Insurance (virtually everybody). Each individual is assigned a unique identification number.

Up to three hospital diagnoses are coded according to the ICD Revision operating at the time of the discharge. Histological diagnoses are computerised in several, but not all LHUs. An effort has been made by RTV during this pilot study to complete the computerisation of cancer diagnoses in several pathology departments. Diagnoses are mostly coded according to SNOMED or, in a few hospitals, to ICD-01.

The feasibility study started in the second half of 1990 and covers data collection for the period 1987-89. The period was chosen because of the availability of computerised data and the potential for cross-linkage with the computerised files for a variable number of years (2-8) available before 1987. This has facilitated the exclusion of prevalent cases.

Between the second half of 1990 and the end of 1992 the computerised death certificates and the computerised files from hospitals and pathology departments were acquired by the RTV from a selected number of LHUs.

The data have been treated and controlled according to the scheme in Figure 1. In particular, the first step of processing includes, in sequence, the preliminary cleaning of the raw data by the exclusion of non-residents, prevalent cases, multiple records, incorrect information, etc.

The computerised population files of residents in the LHU is linked to hospital discharges, pathology reports and death certificates of both the index LHU and of the other LHUs included in the network, thereby allowing cases to be identified even though diagnosed and/or treated outside the LHU of residence. Considering that the main hospital centres of the region are included in the network, the proportion of cases missed by the Registry should be small.

**Figure 1** Venetian tumour registry (RTV) data flow scheme.

All the histological and cytological diagnoses coded in SNOMED are transcoded through an *ad hoc* program developed 'in-house' into ICD codes. The number of computerised pathology records originally processed was 405 521 of which only 0.29% were not transcoded by the program and consequently were manually processed.

The data are then entered into the RTV database management system using the package Statistical Information Retrieval (SIR) installed on the VAX mainframe of the University of Padua.

After further logical quality control checks, the data are processed by a SIR subroutine, which reads the ICD codes available for each subject (hospital, pathology, death certificate) and, whenever possible assigns a Registry diagnosis (three digits, ICD IX) according to an algorithm which accepts: (1) cancer cases with full concordance between two or more sources; (2) histologically confirmed cases with at least one concordant or compatible (e.g. metastases or ill-defined) hospital discharge or death certificate; (3) histologically confirmed skin cancer (173) unless in combination with skin melanoma (172); and (4) histologically confirmed benign, *in situ* and uncertain behaviour tumours.

Diagnoses based on one source only are systematically rejected and manually checked unless based on pathology reports. Multiple primary tumours are also checked. Manual checking is obviously required also for all discordant or incompatible diagnoses according to the criteria reported above.

Individual checks are first performed by using as far as possible the computerised databases of the LHUs, which contain more information than those routinely collected by the Registry (e.g. from radiology departments, laboratory archives, etc.), and then, if necessary, by direct examination of the information from the hospital records.

We present here the final results from the pilot study which has been completed in nine LHUs out of 11 originally included. In two LHUs an important under-registration of cases was suggested, probably due, in part, to treatment of residents of peripheral areas in hospitals in areas not covered by the RTV. The total population at the 1991 census was 1 449 513 corresponding to 33.1% of the total regional population.

Cases with a new diagnosis of cancer between 1 January 1987 and 31 December 1989 have been included in incidence estimates. The earliest year for which computerised files were available was 1979 for hospital discharges and 1984 for pathology reports. Death certificates concerned, of course, only residents who died during the study period.

The total original computerised material received from the nine LHUs consisted of 623 562 hospital discharges, 405 521 pathology records and 35 790 death certificates.

### Results

After exclusion of subjects without cancer, non-residents and multiple records of the same disease for the same patient, the total number of subjects with a cancer diagnosis from any source during the study period was 67 638, of which 34 057 (50.4%) subjects for whom a diagnosis of cancer for the same site before 1 January 1987 existed.

After logical checks and quality control, 33 581 subjects have been treated by a SIR subroutine (SITE) created *adhoc* by RTV which checks the consistency and compatibility of the diagnoses and the ICD codes. The data flow is shown in Figure 2. For 22 087 of these cases (61.3%) a coded diagnosis was assigned by the program while about one-third of the cancer patients needed further confirmation, which was achieved for 10 298, whereas 1196 were excluded because at further checks they were found to be non-tumours, duplicates or non-residents.

The proportion of computer-assisted case resolution presents a large inter-LUH variability, ranging from 50% to 80%. This is mainly owing to the variable proportion of cases with hospital records only, which is larger in the LHUs where the computerisation of the archives took place more recently, thus resulting in a predominantly manual search of prevalent cases.

For 2972 subjects, 8.9% of the total reported incident cases or 25.9% of the rejected cases, it was necessary to examine the original hospital records, whereas for all the other rejected subjects it was sufficient to search for further information in the computerised databases of the LHUs involved.

The total number of subjects with confirmed incident tumours (including *in situ* lesions) during the period 1987–89 was 32 385, of whom 20 919 had malignant and 11 466 benign, *in situ* and uncertain behaviour tumours. These figures correspond to 21 382 malignant cancer cases, and to 12 921 benign, *in situ* or uncertain behaviour cancer cases owing to 463 multiple primary tumours in the first group and 1455 in the latter.

Computerised data on benign tumours (210–239) was available from the pathology departments only for about 42% of the population under study.

Table I gives in detail the distribution of confirmed incident malignant tumours by source or combination of sources of information.

Seventy-eight per cent of male cases and 76% of female cases are diagnosed on the basis of the combination of different and independent sources. The proportion of cases with a microscopic confirmation is close to 80% for both males and females, whereas 16% and 15% of cases, among males and females respectively, had a diagnosis of cancer in the hospitals admissions without histological or cytological confirmation. Overall, the data appear to be consistent across the sexes apart from the slightly higher proportion of death

**Figure 2** Venetian tumour tegistry (RTV), pilot study 1987–89, data flow.

**Table I** Distribution of confirmed malignant tumours 140–208 by source and combination of sources of information

| *Source or combination of sources* | *Males* n | % | *Females* n | % |
|---|---|---|---|---|
| Death certificate only | 455 | 3.9 | 467 | 4.8 |
| Hospital only | 624 | 5.4 | 598 | 6.2 |
| Radiotherapy only | 75 | 0.6 | 87 | 0.9 |
| Pathology only | 1479 | 12.6 | 1186 | 12.3 |
| Hospital + death certificate | 1242 | 10.6 | 854 | 8.8 |
| Hospital + death certificate + radiotherapy | 255 | 2.2 | 176 | 1.8 |
| Pathology + death certificate | 175 | 1.5 | 102 | 1.1 |
| Hospital + pathology | 4742 | 40.5 | 4719 | 48.8 |
| Hospital + death certificate + radiotherapy | 190 | 1.6 | 80 | 0.8 |
| Hospital + pathology + death certificate | 2472 | 21.1 | 1391 | 14.4 |
| Total | 11 709 | 100 | 9660 | 100 |

certificate only (DCO) cases among females. The higher curability of important female cancers, like uterine and breast cancers, is reflected by the smaller contribution of death certificates to the group of diagnoses from multiple sources.

Our data do not seem to differ, in terms of quality, from other cancer registries in Italy or, in general, in Europe apart from those established over a long period in the Nordic Countries (Parkin *et al*., 1992). The slightly lower M/I ratios (0.68 for males and 0.56 for females) may reflect some residual prevalence, particularly for some specific cancer site like, for example, breast, which is difficult to control fully during the initial period of activity of the Registry.

### Discussion

The results from this feasibility study of an automated cancer registration system in the Venetian Region of Italy appear to be reasonably satisfactory.

The present results rely on the availability of independent and computerised sources of information containing health-related information at individual level and the high proportion of cases confirmed by histology is a key condition for assuring an acceptable level of reliability of the computerised and coded diagnoses.

The proportion of cases which require manual checks slightly exceeds one-third of the total number of cases. An important limitation was the large number of prevalent cases, based on hospital discharges only, which had to be ascertained manually during this initial phase. The LHUs with most complete computerised archives tend to have, in general, data of higher quality and reach a proportion of computer-assisted case resolution close to 80%. We believe that this is the feasible quota of computer-assisted diagnoses which can be reached at present in this region of Italy. Such a situation would allow the cancer registration system to be operational over a large population at reduced costs.

The budget during the feasibility study has been 100 000 US dollars per year plus two MD epidemiologists (60%) and two statisticians (80%) with a total personnel cost of approximately 120 000 US dollars per year. The estimated cost per case per year has therefore been close to 19 US dollars (benign tumours included).

Further reduction of the proportion of cases requiring manual checks can be achieved only through the increase of the quota of cases with microscopic confirmation which is now around 80%.

The use of the three (and not the four) digit ICD code in the computer-assisted diagnosis represents an important limitation. This problem has been partially solved by transcoding the information from the T and M codes of the SNOMED system into ICD-O. This is obviously feasible only with cases microscopically defined (i.e. approximately 80%). The transformed data have been verified and included in the EUROCIM network coordinated by the International Agency for Research on Cancer.

An important aspect is the strong inter-relationship of the Cancer Registry with the public health system, of which it reflects the level of quality of the data available and with which it interacts, contributing to the improvement of the data stored by the hospitals and the departments of pathology.

We would also like to stress the value for case resolution that comes from the availability of the full computerised hospital history of admissions and discharges of patients with discordant diagnoses, thus suggesting that priority should be given to the epidemiological use of the existing computerised health archives often considered of mere administrative interest.

Given the above results of acceptable quality so far obtained we are proceeding to the updating from 1990 to 1994 of the RTV database with the hypothesis of a smaller proportion of cases to be manually solved owing to a smaller number of cases based on hospital or death certificate only registered for the first time by the Registry. Should this be confirmed, the Registry could provide incidence data within 2 years of diagnosis. We are also evaluating the possibility of extending the network to other areas of the region.

In addition the availability of a large database (covering half of the LHUs in the present study) on neoplastic diseases diagnosed as benign, *in situ* or of uncertain behaviour, may have potential value.

**Acknowledgements**
The study was partially supported by the Italian Association for Cancer Research (AIRC) and the National Research Council (CNR, contract no. 91.00327.CT04).

### References

CODLING BW, PHEBY D, HAGEN DL AND DUFFIN MF. (1990). Cancer registration by linking pathology and district PAS data. *Br. J. Cancer*, **62**, 271–274.

GREEN M. (1992). *Users' Requirements Study for a System of Patient and Tumour Records of the Ontario Cancer Registry*, Logic Science Systems: Toronto.

JENSEN OM, PARKIN DM, MACLENNAN R, MUIR CS AND SKEET RG. (1991). *Cancer Registration: Principles and Methods.* IARC Publication 95. IARC: Lyon.

MANDERS T AND STORM HH. (1993). Improving the completeness of the Danish Cancer Registry by linkage with the Danish National Hospital Discharge Registry. In *Book of Abstracts*. Annual Meeting of the International Association of Cancer Registries. Bratislava, Slovakia. 13–16 September 1993, p. 116.

MOSS SM, COHN L, MCNICHOL F AND SMITH JAE. (1993). A Computerised Registry Enquiry System. In *Book of Abstracts*. Annual Meeting of the International Association of Cancer Registries. Bratislava, Slovakia. 13–16 September 1993, p. 112.

MUIR C, WATERHOUSE J, MACK T, POWELL J AND WHELAN S. (1987). *Cancer Incidence in Five Continents.* Vol V. IARC Publications 88. IARC: Lyon.

PARKIN DM, MUIR CS, WHELAN SL, GAO YT, FERLAY J AND POWELL J. (1992). *Cancer Incidence in Five Continents.* Vol VI. IARC Publication 120. IARC: Lyon.

PHEBY DFH AND CODLING DW. (1993). Developing links with histopathology computer systems for cancer registration. In *OPCS, Annual Reference Value: Cancer Incidence and Mortality 1988 (England and Wales)*. HMSO: London.

PUKKALA E. (1992). Use of record linkage in small-area studies. In *Geographical and Environmental Epidemiology Methods for Small-area Studies.* Elliott E, Cuzick J, English D, Stern R. (eds), pp. 125–131, Oxford University Press: Oxford.

SIR Inc. Scientific Information Retrieval. (1993). *Data Base Reference Manual, Version 3.2.* SIR: Sydney.

TEPPO L, PUKKALA E AND LEHTONEN M. (1994). Data quality and quality control of a population-based cancer registry. Experience in Finland. *Acta Oncologica*, **33**, **4**, 356–369.

WATERHOUSE J, MUIR C, SHANUGARATNAM K AND POWELL J. (1982). *Cancer Incidence in Five Continents.* Vol IV. IARC Publication 42. IARC: Lyon.

ZANETTI R AND CROSIGNANI P. (1992). *Cancer in Italy - Incidence Data from Cancer Registries 1983–87.* Lega Italiana per la Lotta Contro i Tumori, Associazione Italiana di Epidemiologia: Turin.